# Physicochemical design rules for the formulation of novel salt particles with optimised saltiness

**DOI:** 10.1016/j.foodchem.2021.129990

**Published:** 2021-10-30

**Authors:** Katherine E. Hurst, Charfedinne Ayed, Ivan N. Derbenev, Louise Hewson, Ian D. Fisk

**Affiliations:** a Division of Food, Nutrition and Dietetics, University of Nottingham, Sutton Bonington Campus, Loughborough LE12 5RD, United Kingdom; b School of Chemistry, University of Nottingham, University Park, Nottingham, NG7 2RD, United Kingdom; c The University of Adelaide, North Terrace, Adelaide, South Australia, Australia

**Keywords:** Sodium reduction, Time-intensity, Sodium dissolution kinetics, Particle adhesion, Foam-mat processing

## Abstract

Novel sodium reduction strategies are urgently required by the food industry. We hypothesised that redesigning salt crystals (size, density, hydrophobicity and flow properties) will offer a new route to increase saltiness and therefore reduce sodium. Eight salts were compared with different physicochemical properties, the resultant particles were characterised and adhesion to product, loss in-pack, rate of dissolution and ultimately saltiness perception were evaluated. Principle findings included that particle adhesion was driven by particle size (r = -0.85, p = 0.008), bulk density (r = -0.80, p = 0.017) and flow properties (r = 0.77, p = 0.015); loss in-pack was associated with particle size and hydrophobicity of the salt particle while dissolution and/or saltiness perception was also driven by particle size and hydrophobicity of the salt particle. The findings offer a new set of design rules for future ingredient design for the food and flavour industries.

## Highlights

• Unique salts with varying physicochemical properties were designed.
• Salts were applied to a model system with a controlled surface area.
• Transfer efficiency was driven by size, density and flow properties.
• Loss from the product in packaging was driven by particle size.
• Dissolution and/or saltiness were driven by size and hydrophobicity.

## Graphical abstract

**Figure:**
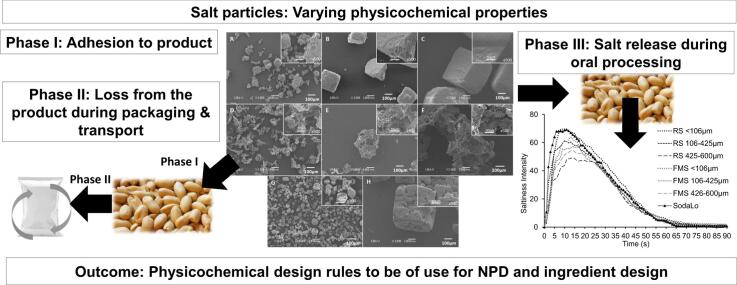

## 1 Introduction

Over-consumption of sodium chloride (NaCl) salt can lead to an increased risk of high blood pressure, cardiovascular disease, stomach cancer and kidney-related disease (Adler, Taylor, Martin, Gottlieb, Taylor, & Ebrahim, 2014). Average NaCl salt consumption exceeds the recommended levels of <5 g NaCl/day (World Health Organisation, 2018), with consumption of >8 g/day, >9 g/day and >12 g/day in the UK, USA and Asia (Public Health England, 2016). As 85% of consumed sodium originates from NaCl in processed foods, there is a high demand for food manufacturers to produce sodium-reduced foods while maintaining flavour profile, appearance and consumer acceptability (Gibson, Armstrong, & McIlveen, 2000). However, sodium is included for structure formation, taste and flavour enhancement, making reduction challenging due to its multifaceted benefits.

To perceive saltiness in foods, NaCl must first dissociate in saliva forming free sodium ions (Na^+^), move close to oral taste receptor cells (TRC) and passively diffuse through epithelial amiloride-sensitive sodium channels (ENaC) on the TRC surface (Chandrashekar, Hoon, Ryba, & Zuker, 2006). This ultimately leads to the transmission of taste signals and saltiness perception (Delwiche & O’mahony, 1996).

In dry snack foods, such as crisps and peanuts, a significant proportion of sodium, in the form of topically applied salt particles, is lost during processing, packaging, transport or storage due to poor adhesion of the dense crystal structures or is consumed without being perceived as swallowing occurs before the salt is dissolved (Tian and Fisk, 2012, Yucel and Peterson, 2015b, Yucel and Peterson, 2015a). To optimise sodium perception, it is imperative to; maximise the fraction of Na^+^ that successfully adheres to the product and delivers to the oral cavity and optimise the rate at which the salt particle dissolves.

We, therefore, propose three key phases for the optimal design of salt particles: Phase I: Adhesion during application and before packaging: Poor adhesion results in unnecessary wastage and directly impacts the heterogeneity of product and sodium levels in-pack; Phase II: Adhesion during packaging and transport: Salt should remain on the product as salt particles not associated with the product are unlikely to be consumed, leading to elevated pack declared sodium levels; Phase III: Release during oral processing: Salt release should occur quickly and remain separate from the bolus to enable effective diffusion of free Na^+^ ions to TRCs.

Redesigning salt particles (particle size, morphology, surface properties and flow properties) has previously been proposed as a route to optimise adhesion, enhance dissolution rate and increase saltiness perception (Chindapan et al., 2018, Moncada et al., 2015, Rama et al., 2013, Vinitha et al., 2020, Yi et al., 2017). In addition to milling (Rama, Chiu, Carvalho Da Silva, Hewson, Hort, & Fisk, 2013), the size and shape of salt particles can also be modified through controlled drying such as spray-drying to produce small NaCl enriched particles (Chindapan, Niamnuy, & Devahastin, 2018) or the addition of sodium ferrocyanide during vacuum crystallisation of brine to produce dendritic salt with a high surface area (Buck and Barringer, 2007, Matz, 2012). Drying of a foamed brine also produces a product with a high surface area (foam-mat drying) (Chokumnoyporn et al., 2016, Rajkumar et al., 2007, Teoh et al., 2016). However, despite studies showing sodium reduction is possible by increasing crystal surface area (Rama et al., 2013, Shen et al., 2015), this is often technically challenging in humid environments due to caking of highly hygroscopic finely milled salt.

Adhesion of salt particles is also key to producing well-seasoned products (Buck & Barringer, 2007). Changing the size or shape of salt particles has been shown to enhance adherence (Buck and Barringer, 2007, Halim and Barringer, 2007). However, adhesion must be reversible, enabling salt release and dissolution during oral processing (Quilaqueo et al., 2015, Rama et al., 2013). Smaller particles adhere more efficiently than larger particles (Buck and Barringer, 2007, Halim and Barringer, 2007), while flakes coated more efficiently than cubic salt particles due to an increased surface area (Miller & Barringer, 2002). Finding a salt crystal morphology that provides strong adhesion whilst achieving effective sodium delivery to the oral receptor is critical in searching for a reduced salt alternative for topical application (Rama et al., 2013, Shen et al., 2015).

Due to the multifaceted role of salt in food and the wide-ranging interacting factors that impact its efficacy (Phase I, II and III), it is unlikely that a single pronged approach will result in the successful development of novel salt particles. We, therefore, explored the potential of modifying a range of physicochemical properties simultaneously (particle size, density, hydrophobicity and flow properties) on adhesion to product, loss in-pack, rate of dissolution and saltiness perception. Ultimately the aim is to generate a series of design rules for novel salt particles and inform ingredient design for future product development for food, flavour and other industries.

## 2 Materials and methods

Eight diverse salt samples produced using a range of processing methods were evaluated. Their physicochemical properties, the efficiency of transfer to product and adhesion to product, the release of sodium ions during dissolution and subsequent saltiness perception was assessed.

### 2.1 Formulation of model salt particles

Regular salt (RS) (Sainsbury's, London, UK) was milled using a coffee grinder and mechanically sieved using nickel sieves (Fisher, Loughborough, UK) into three different fractions: <106, 106–425 and 425–600 µm. Samples are referred to as RS plus the sieve sizes used.

SODA-LO® Salt Microspheres Extra Fine salt (Tate and Lyle, London, UK) was formed by spray-drying a salt solution with maltodextrin and has a lower density/higher bulk porosity than RS. Dendritic salt has a modified surface area and was purchased from Madar Corporation Ltd, Hampshire, UK.

Foam-mat salt (FMS) has a lower density/higher bulk porosity than RS crystals. Three fractions varying in particle size, were prepared by blending commercial NaCl salt (Sainsbury's, London, UK) in ultrapure water with a hydrophobic egg albumen powder (MyProtein, Cheshire, UK) and methylcellulose (Special Ingredients, Chesterfield, UK) followed by grinding and sieving. The process is further outlined in 2.1.1.

#### 2.1.1 Preparation of foam-mat salt particles

Method and formulation for FMS particle preparation was developed in preliminary experiments (data not published). A solution of 21.7% NaCl, 5.4% egg albumen powder, 0.36% methylcellulose and 72.5% ultrapure water was stirred with a magnetic stirrer at 1000 rpm with a constant temperature (5 °C for 15 h). After mixing, the solution was foamed using a Kenwood Chef mixer (Kenwood Limited, Havant, UK) with a stationary bowl and whisk attachment at room temperature (17–18 °C) for 8 mins on speed setting 6 to form a stable gas–liquid foam. The resultant stable foam was spread over a baking sheet at a thickness of <5 mm and dried in a fan convection oven at 60 °C until it reached a constant weight. Once dry, the thin porous honeycomb structure was scraped from the tray and ground using a mortar and pestle and mechanically sieved using nickel sieves (Fisher, Loughborough, UK) to standardise the particle size into 3 fractions (<106, 106–425, 425–600 µm). These samples are referred to as FMS and mechanically sieved sizes.

### 2.2 Physicochemical characterisation of model salt particles

#### 2.2.1 Morphological characterisation

Morphological observations of the salt particles were made using a JEOL 6060LV Scanning Electron Microscope (SEM) (JOEL Ltd, Tokyo, Japan) at 10 kV for isolated salt particles (Rama, Chiu, Carvalho Da Silva, Hewson, Hort, & Fisk, 2013).

#### 2.2.2 Moisture content and water activity

Moisture content (MC) and water activity (a_w_) measurements were based on methodologies outlined in (Yu, Macnaughtan, Boyer, Linforth, Dinsdale, & Fisk, 2012). The MC of samples was determined gravimetrically by oven drying at 105 °C for 24 h (Memmert GmbH and Co. KG, Schwabach, Germany). MC was calculated using Equation 1.

(1) $$\mathrm{MC}\;\left(\%\right)=\frac{\mathrm{Initial}\;weight-dry\;weight}{\mathrm{dry}\;weight}\times 100$$

A_w_ was analysed using the AquaLab water activity meter (METER Group, Munich, Germany). For A_w_ measurements, samples were placed in a standard A_w_ container with a lid and parafilm wrap to seal the container (samples filled just under half of the sample container, as per manufacturer’s instructions). Samples were left to equilibrate in the sealed containers for 3–4 h at 20 °C (room temperature) before analysis.

#### 2.2.3 Bulk density and tapped density

The bulk density (ρb) of salt samples were obtained gravimetrically using a dry glass 10 mL graduated cylinder at 20 °C (room temperature) and was calculated using the weight and corresponding volume according to Equation 2. The tapped density (ρt) was measured in the same way, but the measuring cylinder was tapped strenuously until no further change in volume took place (Basu & Athmaselvi, 2018).

(2) $$\rho b\;or\;\rho t\;\left(g/mL\right)=\frac{\mathrm{Mass}\;of\;powder\;(g)}{\mathrm{Volume}\;of\;powder\;(mL)}$$

#### 2.2.4 Carr’s Compressibility index (%)

Carr’s Compressibility Index (CI%) of the salt particles was evaluated using the relationship between tapped and bulk densities of the samples (Basu & Athmaselvi, 2018) and expressed as a percentage (Equation 3).

(3) $$\mathrm{CI}\%=\frac{\rho t-\rho b}{\rho t}\times 100$$

CI% indicates flowability of a powder. A free-flowing powder compacts readily, resulting in a similar bulk to tapped density. A powder that flows poorly has a greater bulk density to tapped density, suggesting a greater number of inter-particle interactions. Fine rough particles or those with complex surfaces are known to flow more poorly, and larger, smoother particles flow more readily and have a higher CI%. In general, particles greater than 250 µm tend to be free-flowing, while those below 100 µm tend to be cohesive. A CI% of <10 represents excellent flow, 11–15% good flow, 16–20 Fair flow, 21–25% passable flow, 26–31% poor flow, 32–39% very poor flow, and >40% very, very poor flow (Carr, 1965). Whilst this is an empirical measure, it offers a rapid tool for powder flow characterisation.

#### 2.2.5 Particle size

Particle size analysis was performed using a LS 13 320 Laser Diffraction Particle Size Analyser equipped with Tornado dry powder system (Beckman Coulter, Brea, California, USA). The Fraunhofer theory was used to determine the mean diameters of the particles, as explained in Soukoulis, Behboudi-Jobbehdar, Yonekura, Parmenter, & Fisk, 2014.

#### 2.2.6 Colour

Salt samples were placed in small plastic cuvettes for analysis. Lightness (L*), redness (a*) and yellowness (b*) were measured by a HunterLab colorimeter, and the whiteness index was calculated using Equation 4 (Chokumnoyporn, Sriwattana, & Prinyawiwatkul, 2016).

$\mathrm{Whiteness}\;Index=100-{\left[{\left(100-L^{*}\right)}^{2}+{a^{*}}^{2}+{b^{*}}^{2}\right]}^{1/2}$

#### 2.2.7 Total sodium content

The following method was adapted from Ayed et al. (2021). Nitric Acid 68% for Trace Metal Analysis (Thermo Fisher Scientific, Waltham, Massachusetts, USA) and ultra-pure water, (Millipore, Bedford, Massachusetts, USA) was used. All salt samples except FMS samples were prepared by dissolving 0.05 g salt into 10 mL of 2% nitric acid and were then diluted by a factor of 100 using the same 2% nitric acid solution and transferred to clean polypropylene inductively coupled plasma mass-spectrometry (ICP-MS) tubes (Sarstedt Inc, Newton, North Carolina, USA). RS, dendritic and SODA-LO® samples dissolved readily in the 2% nitric acid matrix. Foam-mat samples required further digestion steps due to lower solubility and were prepared by adding 10 mL of 68% nitric acid to 0.2 g of FMS in polypropylene digestion tubes (Anton Paar, Graz, Austria), and digested (teflon-coated graphite Block Digestor, Analysco Ltd, Oxford, UK) at 95 °C (2 hr) with polypropylene watch glasses (Anton Paar, Graz, Austria) placed on top to allow for refluxing. After cooling, samples were topped up to 50 mL with ultrapure water. Samples were mixed well, 1 mL of sample was removed from the top, diluted by 100 and transferred to ICP-MS tubes. Multi-elemental analysis of the diluted solutions was undertaken by ICP-MS (Thermo Fisher Scientific, Bremen, Germany). Samples were introduced at a flow rate of 1.2 mL/min from an autosampler (Cetac ASX-520) incorporating an ASXpress™ rapid uptake module through a perfluoroalkoxy (PFA) Microflow PFA-ST nebuliser (Thermo Fisher Scientific, Bremen, Germany). Sample processing was undertaken using Qtegra™ software (Thermo Fisher Scientific, Bremen, Germany) and external cross-calibration between pulse-counting and analogue detector modes were used when required. Internal standards, used to correct for instrumental drift, were introduced to the sample stream on a separate line (equal flow rate) via the ASXpress unit. Internal standards included combinations of Sc (10 µg/L), Ge (10 µg/L), Rh (5 µg/L), Re (5 µg/L) and Ir (5 µg/L). The matrices used for internal standards, calibration standards and sample diluents were 2% nitric acid (Fisher Scientific, Loughborough, UK) with 4% methanol (to enhance ionisation of some elements). Multi-element calibration solutions were prepared at different concentration levels of Ca, Mg, Na and K (0–30 mg/L) from a bespoke external multi-element calibration solution (SCP Science, Quebec, Canada).

Concentration was converted to sodium concentration using dilution factors. High purity sodium chloride (Sigma-Aldrich, St. Louis, Missouri, USA) was used as a reference for 100% sodium chloride, percentage NaCl for each sample was calculated. Samples were measured in triplicate with blank samples to remove any contamination effects. Coefficient of variation for analytical triplicates was 0.2–2.8% indicating high precision.

### 2.3 Salt particles – peanut interactions

#### 2.3.1 Measurement of particle adhesion to lightly oiled peanuts

Salt adhesion properties were determined using a modified method from Buck and Barringer, 2007, Sumonsiri and Barringer, 2011. Salt particles (2 g) were added to unsalted pre-oiled peanuts (100 g). Pre-oiled peanuts were made by mixing 1 g sunflower oil (Sainsbury's, London, UK) per 100 g unsalted peanuts (KP Snacks Limited, Slough, UK). The weight of oiled peanuts was recorded as the weight before coating with salt for each sample (Wt_1_). Each salt sample (2 g) and the appropriate amount of pre-oiled peanuts were weighed (Wt_2_) and mixed in a cylindrical plastic container by hand for 30 s using the same rotating motion to mimic a tumble drum used to coat snack foods. The coated salted peanuts were then placed in a 16 cm × 23 cm packaging pouch made from polyethene terephthalate, aluminium foil and polyethene (Fresherpack Ltd, Huddersfield, UK). The weight of the salted peanuts inside the packaging was recorded as the weight of the sample after mixing (Wt_3_). Transfer efficiency (TE %) was then calculated by equation 5. For the packaging test, packaged peanuts (Wt_3_) were sealed and inverted 10 times. Salted peanuts were then poured out into a separate container, and the weight was recorded as the weight after the packaging test (Wt_4_). Adhesion after packaging test (Ad %) was then calculated using equation 6.

(5) $$\mathrm{TE}\left(\%\right)=\frac{{\mathrm{Wt}}_{3}-{\mathrm{Wt}}_{1}}{{\mathrm{Wt}}_{2}-{\mathrm{Wt}}_{1}}\times 100$$

(6) $$\mathrm{Ad}\left(\%\right)=\frac{{\mathrm{Wt}}_{4}-{\mathrm{Wt}}_{1}}{{\mathrm{Wt}}_{2}-{\mathrm{Wt}}_{1}}\times 100$$

Wt_1_: weight of oiled peanuts before coating with salt
Wt_2_: weight of salted peanuts after coating
Wt_3_: weight of the salted peanut sample after mixing once inserted into packaging
Wt_4_: weight of salted peanut sample after packaging test

#### 2.3.2 Salt particle dissolution kinetics in water from lightly oiled peanuts

Salt dissolution was evaluated by measuring conductivity over time, modified from Rama, Chiu, Carvalho Da Silva, Hewson, Hort, and Fisk (2013). Samples (2 g ± 0.2 g) were placed in a dissolution vessel (4.5 cm diameter stainless-steel tea strainer, Arktek Group Limited, Sunderland, UK) and suspended in RO water (500 mL, 20 °C, stirring at 200 rpm). Conductivity (micro siemens per cm^3^) was recorded every 5 s for 200–300 s using a SevenExcellence pH/Ion/Conductivity meter, 4-pole platinum conductivity probe (inLab 710, 0.01–500 micro Siemens/cm^3^) (Mettler Toledo, Columbus, Ohio, USA). Conductivity was normalised to percentage conductivity over time (s). The area under the curve of dissolution graphs was determined using the trapezoidal rule, and labelled as AUC_diss_, presented without units. Other dissolution parameters were extracted including; initial slope (determined by calculating the gradient of the curve between 0 and 20 s) and time to 25% (T25%), 50% (T50%), 75% (T50%) and 90% (T90%) conductivity, in seconds.

### 2.4 Sensory evaluation of model salt particles

#### 2.4.1 Sensory panel and samples

A sensory panel consisting of 12 screened and highly experienced assessors (3 men and 9 women, aged 48–72 years) assessed final product samples for saltiness intensity. All assessments took place in individual tasting booths designed to meet ISO 8589:2007 standards with red coloured lighting to minimise product appearance differences. Batches of salted peanut samples were prepared using 1.31–1.83 g of model salt (depending on NaCl content) added to 100 g of oiled peanuts to reach a final concentration of 1.3 g NaCl per 100 g of oiled peanuts. Panellists were served 2 g (±0.1 g) of salted peanut sample in small plastics pots. In total, seven of the eight salts underwent sensory assessment (dendritic salt was excluded as it could not be classified as food grade).

#### 2.4.2 Time-intensity (TI)

TI methodology was carried out based on the ASTM, 2017 standard. Before data collection, three 2-hour training sessions took place to familiarise the panellists on the methodology, assessment protocol, saltiness scale, reference samples and test samples. Panellists were instructed to record their perception of saltiness intensity over 90 s by moving a mouse on a linear scale. Data was captured using EyeQuestion software version 4.11.6. Panellists started their saltiness ratings once the sample was placed in the mouth from the pot by clicking start on the screen. Chewing rate and swallowing time were controlled to minimise variation caused by individual differences in their chewing behaviours. Panellists chewed at a rate of 70 beats per minute controlled by the sound of a metronome while simultaneously evaluating saltiness intensity using a continuous line scale, where the left end represented a saltiness intensity of 0 and the right end a saltiness intensity of 100. All panellists swallowed at 25 s and data collection finished at 90 s. Samples were assessed in duplicate by each panellist in a randomised order. Water and unsalted crackers were provided as a palate cleanser, with a 10-minute break between samples. Data from 10 panellists (3 men, 7 women, aged 48–72) were used from the total of the 12 panellists based on consistent performance and attendance.

#### 2.4.3 Extracted TI parameters

A number of parameters were extracted from TI curves relating to saltiness intensity, rate and duration, including perceived maximum intensity of saltiness (Imax), area under the TI curve (AUC_sensory_) and the maximum perceived saltiness over time to Imax (rate Imax). Extracted parameters are further detailed in Fig. 3 and Fig. 4.

### 2.5 Statistical analysis

Data analysis was performed using XLStat Sensory version 2020.1.2. Experiments were performed in triplicate, and mean values reported unless otherwise stated. Differences between samples for each variable were determined using analysis of variance (ANOVA) and Tukey's Honestly Significant Difference (HSD) test, p < 0.05. Average TI curves were constructed by calculating the mean intensity value at each time point across panellists and replicates. The extracted TI parameters were subjected to ANOVA (products, panellists and their interactions as fixed variables), followed by Tukey's HSD. Correlations between variables were determined using the Pearson product-moment correlation coefficient (see also supplementary materials). Data was subjected to Partial Least Squares Regression (PLS-R) to visualise relationships between variables.

## 3 Results and discussion

In this study, a range of salts of varying physicochemical properties and macroscopic flow behaviours were assessed to understand the key drivers behind salt adherence and transfer properties, dissolution kinetics and saltiness perception. Exploration of the morphology of the salt particles and their physicochemical properties will first be discussed, followed by the dissolution properties (the rate at which the salt particles dissolve in model saliva) and then ultimately the influence of these parameters on saltiness perception. Relationships, interactions and main drivers for each of the 3 key phases outlined are discussed considering these findings, concluding with proposed design rules for future product development.

### 3.1 Morphology of model salt particles evaluated by scanning electron microscopy

**Fig. 1:**
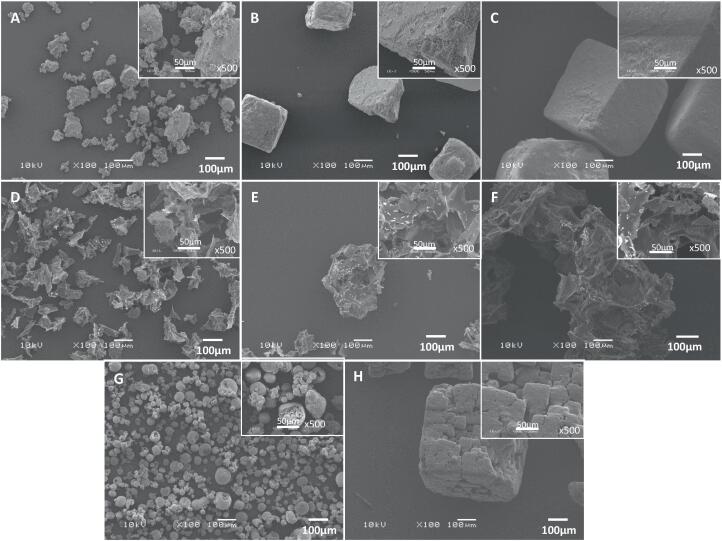
Scanning Electron Microscopy images of model salts: (A) RS < 106 µm, (B) RS 106–425 µm, (C) RS 425–600 µm, (D) FMS < 106 µm, (E) FMS 106–425 µm, (F) FMS 425–600 µm, (G) SODA-LO® and (H) dendritic at magnification × 100 for each of the main images and ×500 magnification images are displayed in the top right corner for each salt sample.

SEM images of salt particles are shown in Fig. 1. Similarly to SEM images presented in Rama, Chiu, Carvalho Da Silva, Hewson, Hort, and Fisk (2013), very fine salt crystals (<106 µm) have an irregular shape due to milling (Fig. 1A). The larger RS samples are dense crystals with smooth topology (Fig. 1B, 1C) that pack closely together due to their smooth flat surfaces (Fig. 1C) and have no internal voids. SODA-LO® particles (Fig. 1G) are smooth pseudospherical structures; these pack loosely and are often damaged or cracked showing internal voids and a higher surface area than RS. The process patent for SODA-LO® (Shen et al., 2013) shows clumped aggregates of microspheres; this can be seen in Fig. 1 where numerous smaller spheres can be found inside the larger SODA-LO® particles (Fig. 1G ×500 magnification). This is similar to other spray-dried products such as fruit powders (Darniadi, Ho, & Murray, 2018). The dendritic salt (Fig. 1H) has an overall cubic shape with surface irregularities resulting in a rough surface topology with a layered appearance, with evidence of small internal voids and a slightly elevated surface area compared to RS. In this study and previous studies (Triyastuti, Kumoro, & Djaeni, 2017), foam-mat powders have a spikey/flake-like structure due to the surface bubbles on the films that are broken up during grinding resulting in a powder that packs loosely with no apparent clumping (Fig. 1D-F). FMS samples appear to have a high surface area and many internal voids at both the ×100 and ×500 magnification.

### 3.2 Moisture content and water activity of model salt particles

MC and a_w_ are presented in Table 1. FMS and SODA-LO® have a slightly higher MC and a_w_. RS samples have a lower MC and a_w_. MC and a_w_ are significantly higher (p < 0.05) in the samples that contain secondary materials, such as maltodextrin in the SODA-LO® sample and egg albumin and methylcellulose in FMS samples. According to the product specification, the MC of SODA-LO® is 0.7% which is close to that measured in this study (0.75 ± 0.03%). A higher MC in these particles is probably due to inefficient evaporation of water from the salt crystal during spray-drying and foam-mat drying (Vinitha, Leena, Moses, & Anandharamakrishnan, 2021) due to water binding with the proteins and sugars. A_w_ measures the amount of water available for biochemical reactions. It can be used as a predictor for the microbial stability of food. A_w_ values in the modified salts were between 0.35 and 0.44, while RS and dendritic salts were between 0.28 and 0.31 and were significantly lower (p < 0.05) than the modified salt samples, except FMS 106–425 µm (Table 1). An increase in MC and A_w_ may have consequences on the long term microbial stability of the salt powder as a product. However, as all the salt samples had an MC of <1% and an A_w_ of <0.5, this is unlikely to be an issue as typically, an a_w_ value of >0.6 is quoted as being required for microbial growth (Barbosa-Cánovas, Fontana, Schmidt, Labuza, & Barbosa-Cánovas, 2007) and these small differences in water content are unlikely to have a significant impact on the physical properties of the samples.

**Table 1:** Physicochemical properties, transfer efficiency and adhesion capabilities (mean ± SD) of different types of salt crystal and modified salt particles. Values in the same row with different letters are significantly different (P < 0.05).

	Salt Particles							
	RS < 106 µm	RS 106–425 µm	RS 425–600 µm	Dendritic	SODA-LO®	FMS < 106 µm	FMS 106–425 µm	FMS 425–600 µm
Moisture content (%)	0.01 ± 0.01d	0.06 ± 0.02cd	0.04 ± 0.02d	0.06 ± 0.03cd	0.75 ± 0.03a	0.35 ± 0.11bc	0.48 ± 0.35ab	0.48 ± 0.10ab
Water activity	0.31 ± 0.01cd	0.30 ± 0.01cd	0.28 ± 0.02d	0.30 ± 0.00cd	0.41 ± 0.02ab	0.44 ± 0.02a	0.35 ± 0.03bc	0.39 ± 0.04ab
NaCl (%)	99.0 ± 1.0a	99.0 ± 1.0a	99.0 ± 1.0a	100 ± 3.0a	93.4 ± 1.0b	58.6 ± 2.0d	73.5 ± 0.0c	76.6 ± 2.0c
Particle Size diameter (µm)	44.3 ± 34	299.6 ± 115.2	542.6 ± 101.4	239.9 ± 106.2	34.5 ± 23.9	60.69 ± 40.1	150 ± 116.1	294.4 ± 147
Bulk Density (g/ml)	0.69 ± 0.02c	1.23 ± 0.03a	1.30 ± 0.02a	1.02 ± 0.01b	0.39 ± 0.01d	0.13 ± 0.01g	0.26 ± 0.01f	0.34 ± 0.01e
Tapped density (g/ml)	1.00 ± 0.08c	1.42 ± 0.05a	1.39 ± 0.01a	1.18 ± 0.01b	0.52 ± 0.02d	0.23 ± 0.05e	0.43 ± 0.02d	0.45 ± 0.02d
CI (%)	30.8 ± 5.7ab	10.4 ± 4.8d	6.8 ± 1.3d	13.0 ± 1.1cd	25.1 ± 2.3bc	41.8 ± 9.0a	41.0 ± 1.3a	24.0 ± 1.5bc
L*	91.85 ± 0.07a	86.35 ± 0.99d	82.45 ± 1.56e	91.07 ± 0.05 abc	91.28 ± 0.28 ab	89.91 ± 0.04 bc	89.28 ± 020c	85.40 ± 0.33d
a*	0.15 ± 0.01a	0.01 ± 0.02c	−0.05 ± 0.01 cd	0.08 ± 0.03b	−0.08 ± 0.01d	−0.55 ± 0.04e	−1.20 ± 0.03f	−1.54 ± 0.05g
b*	0.57 ± 0.03d	0.17 ± 0.05e	0.03 ± 0.10e	0.10 ± 0.02e	2.50 ± 0.06c	8.24 ± 0.09b	9.70 ± 0.15a	9.55 ± 0.28a
Whiteness index	91.82 ± 0.07a	86.35 ± 0.98b	82.45 ± 1.56c	91.07 ± 0.05a	90.93 ± 0.28a	86.97 ± 0.09b	85.49 ± 0.05b	82.48 ± 0.42c
Transfer Efficiency (%)	97.7 ± 1.3a	93.6 ± 4.1bc	91.6 ± 2.9c	98.3 ± 0.6a	98.4 ± 0.6a	98.7 ± 1.7a	98.8 ± 0.3a	97.2 ± 0.1ab
Adhesion after packaging (%)	95.5 ± 0.6a	89.9 ± 4.1b	88.4 ± 1.6b	96.8 ± 0.8a	95.7 ± 0.8a	95.6 ± 1.0a	94.7 ± 1.8a	87.5 ± 2.3b

Samples: FMS = foam-mat salt; RS = regular salt. Colour: L* = lightness level from 0 = black and 100 = white; a* = redness from red (+) to green (-); b*= yellowness from yellow (+) to blue (-). CI% = Compressibility index: <10 = excellent flow; 11–15% = good flow; 16–20 = fair flow; 21–25% = passable flow; 26–31 = poor flow; 32–39 = very poor flow; >40 = very very poor flow.

### 3.3 NaCl content of model salt particles

SODA-LO® and FMS samples had a significantly lower NaCl content when compared to RS and dendritic salt due to the inclusion of non-NaCl components used to create the three-dimensional structures of these salt particles (Table 1). The three FMS samples would theoretically have similar NaCl % as they were manufactured in the same batches before being separated by sieving. However, FMS < 106 µm sample has a lower NaCl % than the other two FMS samples (p < 0.05). A likely explanation for this is that particles of methylcellulose and egg albumen that are smaller than 106 µm in diameter, settle in this fraction, increasing the proportion of other materials. Due to the differences in NaCl levels across all samples, altered amounts of each salt sample was added to the oiled peanuts prepared for sensory evaluation to ensure equivalent NaCl content between samples. Dendritic salt is the purest of all the salts with 100% NaCl as it does not contain any additional anti-clumping agents.

### 3.4 Colour of model salt particles

Whilst sodium concentration is key for saltiness perception, the appearance of salt particles is essential for consumer acceptability, as consumers expect topically salted snack products to be coated in white or slightly clear salt crystals. Colour properties are, therefore, outlined in Table 1. There is a strong negative correlation between whiteness and particle size (r = -0.84, p = 0.009) due to the increased compactness. The largest particle fractions (FMS and RS 425–600 µm) have significantly lower whiteness indices (p < 0.05) compared to all other samples. SODA-LO®, dendritic and RS 106 µm have a significantly higher whiteness value than the other salts (p < 0.05). The a* values for all samples are all very close to 0, showing no red or green contribution to colour. FMS samples all have values over 8 for b*, meaning these samples have a slight yellow hue (+b*) due to the addition of egg albumen powder (Katekhong & Charoenrein, 2017). In this study, colour differences were minimised during sensory assessment using red booth lights, however, increasing yellowness of samples could impact consumer acceptance.

### 3.5 Tapped density, bulk density and Carr’s Compressibility index of model salt particles

Tapped density, bulk density and CI% are calculated for powders as indicators of ease of reconstitution, packaging, transportation, storage and processing (Marques, Borges, de Mendonça, de Barros Fernandes, & Menezes, 2014). Tapped density and bulk density (Table 1) positively correlated to each other (r = 0.99, p=<0.001). Both are also highly negatively correlated with CI% (r = -0.86, p = 0.006 and r = -0.91, p = 0.002, respectively).

The size of RS directly impacted bulk density and tapped density, with larger particle fractions having higher densities (Table 1). This is expected as larger particles flow and pack more readily. This is also observed for CI%, where the larger particles have a CI% of <11%, indicating “excellent” or “good” flow, the smallest RS (RS < 106 µm) has a CI% of 30.8%, which indicates “poor” flow properties.

Dendritic salt had a bulk density, tapped density and CI% that in all cases is similar or sits between RS < 106 µm and RS 106–425 µm. Given that dendritic salt has a mean particle size (239 µm) that also sits between these two fractions (44 µm and 299 µm), it can be assumed that it behaves similarly to RS particles.

SODA-LO® and FMS particles have lower bulk and tapped densities than all other samples. Whilst SODA-LO® (CI% of 25.1%) has “passible” flow properties, which is similar to RS < 106um (CI% 30.8%), FMS has a high CI% indicating “very very poor” flow properties for FMS < 106 µm and FMS 106–425 µm, and only “passible” flow for FMS 425–600 µm. This indicates a marked difference in powder properties and flow behaviour for the FMS samples compared to RS, dendritic and SODA-LO®. Differences are assumed to be due to the very low bulk density and complex surface geometry formed due to air incorporation during the drying process for SODA-LO® and the inherent structure of dendritic salt particles.

### 3.6 Adhesion and transfer efficiency of model salt particles

#### 3.6.1 Transfer efficiency of model salt particles

Transfer efficiency and particle diameter are negatively correlated (r = −0.85, p = 0.008). ANOVA results (Table 1) show that RS 106–425 µm and RS 425–600 µm samples were significantly lower in transfer efficiency than all other samples, except FMS 425–600 µm, which was not significantly different to RS 106–425 µm. Our results show that reducing the particle size of regular table salt increased transfer efficiency during coating. This supports previous findings by Miller and Barringer, 2002, Sumonsiri and Barringer, 2011. However, despite the change in particle size within the three FMS samples, the level of transfer efficiency is not significantly different, suggesting that both FMS processing and a reduction in particle size may increase transfer efficiency.

Adhesion is the main factor determining the coating efficiency of the peanuts and is mainly due to the viscous oil holding salt particles via liquid bridges formed through capillary forces (Takenaka, Ogata, Yabe, Yamauchi, & Kato, 2006). Miller & Barringer, 2002 explained that finer particles have a smaller mass and therefore have improved adhesion initially as gravity has less effect than for larger masses. Whereas there is a more significant effect of gravity on larger particles, counteracting the total adhesion force, causing less coating. When comparing the same fraction sizes, e.g. RS 106–425 µm and FMS 106–425 µm, there was significantly higher transfer efficiency (P < 0.05) in the salt processed using foam-mat drying (Table 1). The same can be said for fraction size 425–600 µm but not the <106 µm fraction, possibly due to the slightly smaller mean particle size of the FMS samples (Table 1). The improved transfer efficiency of FMS could therefore be, in part, also due to the reduction in density. A decrease in density contributes to the rise in transfer efficiency with correlation values of −0.76 to −0.81 (p < 0.05). CI% is also significantly positively correlated to transfer efficiency (r = 0.77, p = 0.015). Whilst it is hard to separate correlation and causality, our findings indicate that particles with poor free flowing properties are likely to have a higher transfer efficiency, suggesting that not only particle size and density, but also surface properties (i.e. how the particles mutually interact) may play a role in transfer adhesion.

#### 3.6.2 Adhesion of model salt particles after packaging

Poor seasoning and salt adhesion efficiency can be problematic once products are packaged. Particles become unattached and drop to the bottom of the packaging resulting in loss of potential flavour. In this study, all peanut samples lost some surface salt within the packaging. Samples: RS 106–425 µm, RS 425–600 µm and FMS 425–600 µm, all had significantly lower (p < 0.05) adhesion after the packaging test compared to the other samples (Table 1). Similarly to transfer efficiency results, smaller particle sizes remain adhered to the peanuts with less particle loss. Whilst the global correlation between mean particle diameter and adhesion after packaging (r = 0.60) was weaker than transfer efficiency and was not significant (p = 0.11, Table 1), there was a significant impact of particle size on adhesion losses for RS (p < 0.05) and FMS (p < 0.05).

As mentioned previously in section 3.6.1, adhesion forces between particles and food surface are made up of capillary forces due to the presence of oil. Particles are lost from the surface when the external influences are strong enough to split the capillary bridges. Differences in adherence can be interpreted via two different mechanisms. Firstly, friction between peanuts and packaging, and secondly, the impact of the peanut colliding with the bottom of the packet due to gravity and resulting in the loss of salt crystals from the peanut surface. In the first instance, larger salt crystals are more exposed to mutual contact than smaller particles, so the larger particles in this study are lost first. Larger particles are more likely to pack more closely. When coated in fat, they cling to each other, thereby further overcoming adhesion forces.

In the second instance, as the peanuts fall and impact the bottom, they 'shake' off some of the salt particles. This is due to the transfer of kinetic energy from peanuts to salt crystals. The kinetic energy of a salt particle is proportional to its mass and hence its volume. Therefore, larger particle sizes achieve greater kinetic energy than smaller particles with the same density. If this kinetic energy is greater than the adhesion energy, then particles detach from the peanut. The adhesion energy is assumed to be proportional to the contact surface area (Halsey & Levine, 1998). This contact surface area can be estimated as the surface area of one of faces of the salt particles. Therefore, the ratio between kinetic and adhesion energy is proportional to the ratio of salt particle volume and the area of its contacting surface. For larger particles (if their shape is similar to smaller particles), this ratio is greater than for smaller particles, so they detach more easily. Manufacturers aiming to reduce cost and reduce the loss of coating materials could therefore decrease particle size. This study demonstrates a new method of assessing salt transfer and adhesion, taking into account forces incurred during packaging and transport.

### 3.7 Dissolution kinetics of model salt particles

**Fig. 2:**
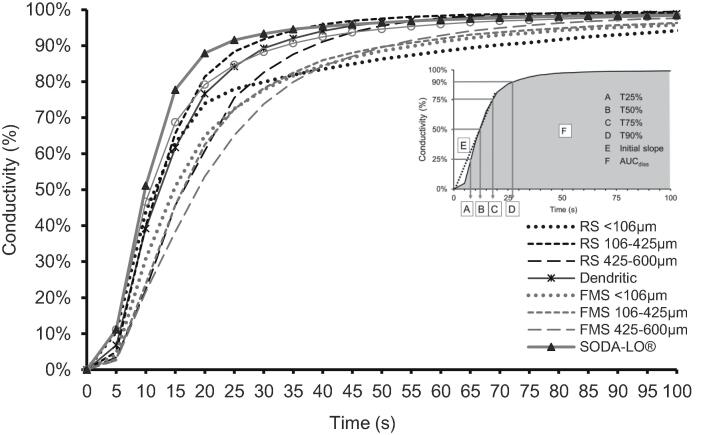
Dissolution curve presented as percentage conductivity of dissolution media (RO water) after immersion for each model salt sample when applied to oiled peanuts contained in a dissolution vessel at 0 s at a constant temperature of 20 °C.

Oral processing is a rapid event. In most cases, salt crystals cannot fully dissolve before a bolus is formed and swallowed (Tian & Fisk, 2012). This incomplete dissolution limits potential saltiness perception. To evaluate this, the salt particles were applied to a real food matrix, oiled peanuts, and dissolution of salt was observed by the change in conductivity of the dissolution media (RO water). Raw conductivity data was converted to a percentage of total conductivity to observe comparative dissolution kinetics between samples over time. The dissolution graph in Fig. 2 shows a slow increase in conductivity (%) in all samples until 5 s and then followed by a rapid increase in conductivity (%) between 5 s and 20 s. After 20 s, the increase in conductivity slows again.

Significant differences were found between the salts for all extracted dissolution parameters (supplementary material 1). SODA-LO® had a significantly higher (p < 0.05) initial dissolution slope than all other samples (4.8% increase per second), except for RS 106–425 µm (4.4% increase per second). SODA-LO® took the shortest time to reach 25, 50, 75 and 90% conductivity, while FMS 425–600 µm required the longest time to reach these same points (except for time to 90% where <106 µm was slowest).

#### 3.7.1 Relationship between salt properties and dissolution kinetics

**Fig. 3:**
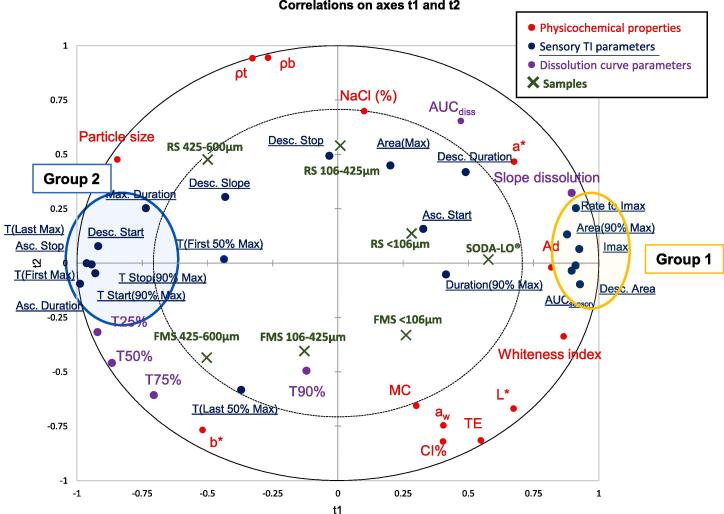
Partial Least Square Regression projections of model salt samples (found in green). The physicochemical and adhesion properties are in red (X), *in vitro* dissolution parameters in purple (X) while sensory variables (Y) are projected in blue. (For interpretation of the references to colour in this figure legend, the reader is referred to the web version of this article.)

Due to the complexity of the various interacting factors, the experimental results from the physicochemical characterisation of samples and *in vitro* dissolution data underwent PLS-R to elucidate relationships between variables (Fig. 3). In general, samples can be seen to be separated on the biplot by particle size along the axis t1 and by NaCl content and processing type on axis t2.

Extracted parameters from the dissolution study are clearly separated on axis t1. T25%, T50% and T75% are closely correlated and are negatively presented on axis t1. T90% is less well resolved. This is partly due to the dissolution kinetics of RS < 106 µm sample, which follows a different dissolution profile, as shown in Fig. 2. The dissolution for this sample slows more quickly than the other samples. This is proposed to be due to the strong adhesion forces between surface oil and the highly compact salt particles of small particle size.

Particle size is positively correlated with the time to reach 25% conductivity (r = 0.65), and this was almost significant at p = 0.06. The linear regression is weaker than expected due to the outlying trend of RS < 106 µm mentioned previously. ANOVA results confirmed significant differences between different particle sizes for curve parameters (further information can be found in supplementary material 1). Samples on the negative side of axis 1, RS and FMS 425–600 µm, can be described as dissolving more slowly due to their larger particle size and lower surface area.

FMS samples are presented closely to T25%, T50% and T75%, indicating that foam-mat drying creates particles that dissolve more slowly. This restricted dissolution in the FMS samples is proposed to be due to the hydrophobic egg albumen and methylcellulose encapsulating the salt and slowing the rate at which it can dissolve. Sodium ions can also chemically interact with negatively charged amino acids within the protein. This binding of free Na^+^ will reduce sodium ion mobility and further slow release and dissolution (Mosca, Andriot, Guichard, & Salles, 2015). This can be supported by solubility values (the degree that a compound can dissociate in water). Solubility values for egg albumen and methylcellulose are 50 mg/ml and 20 mg/ml respectively (Sigma-Aldrich, 2020b, Sigma-Aldrich, 2020c) which are substantially lower than sodium chloride alone (358 mg/ml) (Sigma-Aldrich, 2020a). While this limits FMS use for topical applications, it may benefit product applications that demand a slow sodium release. One example may be an encapsulated salt in bread. It has been previously shown that an inhomogeneous distribution of salt or 'salty spots' within bread can compensate for a reduction in salt. This concept has been previously demonstrated using a fat enrobed salt offering significant sodium reduction potential in bread (Noort, Bult, & Stieger, 2012). However, FMS contains protein rather than fat, which could offer a nutritional benefit. The approach warrants further exploration.

### 3.8 Sensory evaluation of model salt particles

**Fig. 4:**
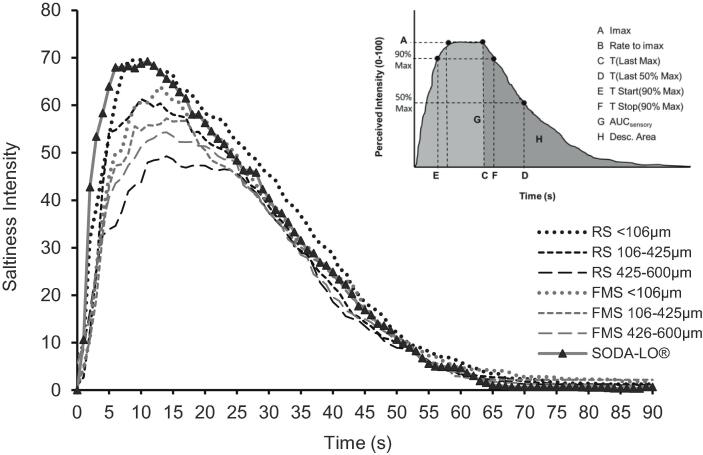
Time-intensity curves averaged across panellists and replicates for perceived saltiness intensity of model salts applied to slightly oiled peanuts with equivalent sodium chloride levels (1.3% in the final product).

Temporal saltiness perception was assessed by TI and average TI curves for each salt are shown in Fig. 4. All curves show a similar curve profile; initial increase in saltiness to a peak, followed by a plateau and gradual decrease until saltiness is no longer perceived, although differences in peak, plateau and time can be observed between samples.

TI curve parameters were extracted and included for PLS-R, identified in blue (Y variables) on the PLS correlation circle (Fig. 3). TI curve parameters; desc. duration, desc. slope, desc. stop, T (First 50% Max), T(Last 50% Max), asc. start, area(max) are all found in the inner circle of Fig. 3, indicating that these parameters are not significantly correlated with the X variables or the samples. These parameters showed no significant differences between samples and were not presented in Table 2; however, due to their importance in understanding the complex interactions of dissolution kinetics and saltiness perception, they are included in the PLS-R. Samples differed significantly in the following curve parameters; Imax, rate to Imax, T(Last Max), AUC_sensory_, T Start (90% Max), T Stop(90% max) and Desc. area and are presented in Table 2.

**Table 2:** Parameters extracted from time-intensity (TI) curves for perceived saltiness (mean ± standard error) which showed significant differences between samples. Values in the same row with different letters are significantly different (p < 0.05).

	Salt Particles						
TI Curve parameters	RS < 106 µm	RS 106–425 µm	RS 425–600 µm	SODA-LO®	FMS < 106 µm	FMS 106–425 µm	FMS 425–600 µm
Imax	77.1 ± 2.6 A	66.9 ± 3.1 ABC	56.4 ± 3.4C	75.6 ± 3.2 A	67.5 ± 3.0 AB	62.9 ± 2.9 BC	59.3 ± 3.1 BC
Rate Imax	11.5 ± 1.8 AB	8.91 ± 1.9 AB	7.34 ± 1.8 AB	13.7 ± 2.5 A	9.47 ± 2.3 AB	6.62 ± 2.0B	5.96 ± 0.67B
T (Last Max)	16.8 ± 1.6 AB	18.0 ± 1.8 AB	20.2 ± 1.9 A	14.6 ± 1.9B	18.0 ± 1.6 AB	17.7 ± 1.6 AB	19.4 ± 1.6 A
AUC_sensory_	2370 ± 176 A	2010 ± 188 ABC	1750 ± 180C	2250 ± 190 AB	2110 ± 167 ABC	1950 ± 186 ABC	1870 ± 156 BC
T Start (90% Max)	6.35 ± 0.78CD	8.55 ± 1.1 ABC	10.4 ± 1.2 A	5.25 ± 1.2 D	7.65 ± 0.93 BCD	9.20 ± 1.1 AB	9.85 ± 1.0 AB
T Stop (90% Max)	18.5 ± 1.8 AB	19.5 ± 2.0 AB	22.2 ± 2.1 AB	17.2 ± 1.9B	19.6 ± 1.8 AB	20.6 ± 2.0 AB	21.0 ± 1.7 AB
Desc. Area	1460 ± 140 A	1140 ± 140 ABC	883 ± 120C	1430 ± 150 AB	1230 ± 120 ABC	1150 ± 130 ABC	1000 ± 120 BC

Samples: FMS = foam-mat salt; RS = regular salt. Time-intensity parameters: Imax = maximum perceived sensory saltiness; Rate Imax = the maximum perceived saltiness over time to Imax; T(Last Max) = time to last highest perceived saltiness value; T(Last 50% Max) = time taken to reach 50% of maximum perceived saltiness; Max. Duration = time at maximum intensity; T Start (90% Max) = time taken to reach the first 90% of maximum perceived saltiness; T Stop (90% Max) = time taken to reach 90% of maximum perceived saltiness on the descent slope of TI curve; AUC_sensory_ = area under the curve of the TI curve; Desc. Area. = the area of the declining slope of the TI curve.

TI parameters are separated by PLS-R along axis t1 (Fig. 3) and are grouped into two clusters: group 1: Rate to Imax, area (90% max), Imax, desc. area, and AUC_sensory__)_ and group 2: (T (first max) and T(last max), T Start(90% Max) and T Stop(90% max), Asc. Stop and max. duration). In general, group 1 is related to the intensity of saltiness and total saltiness. Group 2 is related to the temporal aspects of saltiness perception(. These two groups are highly negatively correlated.

SODA-LO® followed by < 106 µm samples of both RS and FMS are positively correlated with group 1 TI parameters (Fig. 3) with the highest peak intensity of mean TI curves (Fig. 4). ANOVA on extracted parameters from the TI curves confirmed that these salts have the highest values for group 1 parameters (Table 2). In comparison, 425–600 µm samples of both RS and FMS resulted in the lowest mean TI curve peaks (Fig. 4) in saltiness intensity (Table 2) and are positioned further away from group 1 parameters in the PLS-R biplot (Fig. 3). This supports previous studies showing a reduction in particle size results in a higher Imax (Rama, Chiu, Carvalho Da Silva, Hewson, Hort, & Fisk, 2013) and the hypothesis that SODA-LO® with its low density and hollow structure containing internal voids (Fig. 1) would be likely to dissolve much more rapidly than larger particles or those that consist of a more dense crystalline structure (RS).

FMS samples have a less compact structure than RS particles and greater surface area, with more exposed voids (Fig. 1A-F) and would be expected to hydrate more quickly than their size equivalents in RS particles. However, FMS samples of equivalent size to RS particles did not significantly increase in group 1 saltiness parameters. The act of processing is orthogonally presented on the PLS-R with separation on t2, corresponding with the previously presented dissolution data. It is assumed to result from a combination of the encapsulation or binding of Na^+^ by proteins, as explained in 3.7.1, and hydrophobic interactions of salt particles with surface fat which restricted dissolution rates in saliva.

A direct comparison of the equally sized foam-mat particle (FMS < 106 µm) and SODA-LO® suggests that when comparing the two particles with similarly high levels of internal voids and low densities, that the hydrophobic proteins in the FMS samples are binding to the Na^+^ and changing the hydrophobicity of the particle which ultimately restricts Na^+^ release and dissolution (Supplementary Material 1).

#### 3.8.1 Relationship between dissolution kinetics and sensory perception

Dissolution and TI curve parameters showed significant correlations (Supplementary Material 2). The initial slope gradient extracted from between 0 and 20 s of the dissolution curves is positively correlated with rate to Imax (r = 0.85, p = 0.01), which is a key marker of group 1. Furthermore, the initial dissolution slope is also positively correlated with area (90% Max) (r = 0.75, p = 0.062), Imax (r = 0.68, p = 0.087) and AUC_sensory_ (r = 0.59, p = 0.12), although it should be noted that these correlations were not found to be significant (p > 0.05). Dissolution slope is significantly inversely correlated with the group 2 sensory parameters, T(first max) (r = -0.77, p = 0.026), T Start(90% max) (r = -0.77, p = 0.026) and T Stop(90% max) (r = -0.74, p = 0.036). Overall, this suggests that the *in vitro* method can be used as a proxy for the group 1 and group 2 saltiness attributes.

It can, therefore, be shown that samples with a higher dissolution slope value will take less time to reach peak intensity in sensory trials, which, when aiming for salt reduction, is a desirable insight. Whilst AUC_diss_ and T90% from the dissolution data did not show significant correlations with TI curve parameters (p > 0.05); time to 25%, 75% and 50% conductivity are significantly correlated to sensory data (further detailed in bold in Supplementary Table 2). T25% has higher correlation values and lower p-values than T50% and T75%, not unexpected given consumption lasts a relatively short time. After the initial Na^+^ release, other factors come into play, such as saliva flow, clearance, and taste adaptation; therefore, dissolution parameters extracted at relatively longer times do not strongly represent real-life consumption. We conclude that dissolution slope and time to 25% are the best predictors for predicting saltiness perception. In this study, it is also important to note that assessment of saltiness intensity was performed following a clearly defined protocol that standardised oral processing, thereby minimising variation in perception due to eating behaviour. This should be addressed in future studies with product consumers using free eating paradigms.

Other studies have also shown strong correlations between dissolution rates and TI curve parameters using artificial saliva as the *in vitro* dissolution media (Vella, Marcone, & Duizer, 2012). Our current study used RO water as a dissolution media. It successfully predicted sensory outcomes suggesting RO water could be used as a simple alternative to the time and cost expense of artificial saliva if dissolution is the primary experimental aim.

It is noteworthy that whilst the *in vitro* sodium dissolution method used in this study was able to predict defined saltiness TI parameters, multiple factors relate to eating behaviour such as; oral processing and mouth behaviour, saliva composition, salivary flow rates, bolus clearance rates, taste adaption and chewing patterns and therefore should also be considered for investigation in future studies.

## 4 Conclusions

A range of model salt particles which varied in size, density, hydrophobicity and flow properties were used to explore the impact of particle design on adhesion to product, loss in-pack, rate of dissolution and saltiness perception, ultimately to generate a series of design rules that address each of the initial three phases proposed as potential routes to optimise saltiness perception.

**Phase I: Adhesion during application and before packaging:**

Key Finding: Transfer efficiency is driven by particle size (r = -0.85, p = 0.008), bulk density (r = -0.801, p < 0.05) and flow properties (r = 0.77, p = 0.015).

• Decreasing regular table salt particle size increased transfer efficiency during coating, likely due to increased interaction with surface fat on the product.
• Foam-mat processing increased transfer efficiency indicating this is due to reduced bulk density.
• Flow properties were correlated with transfer efficiency suggesting particle–particle interactions also play a role.

**Phase II: Adhesion during packaging and transport:**

Key Finding: Loss from the product in packaging is driven by particle size (p < 0.05).

• Smaller particle sizes exhibited less loss due to enhanced adhesion energy between surface oil on the product and the smaller salt crystals.

**Phase III: Release during oral processing:**

Key Finding: Dissolution and/or saltiness are driven by particle size (p < 0.05) and hydrophobicity.

• Smaller particles sizes were associated with faster sodium dissolution rates; however, this was compromised for highly dense small particles due to high levels of interaction with surface fats.
• Smaller particle sizes had a greater saltiness intensity (Imax) due to faster dissolution in saliva.
• Greater particle hydrophobicity resulted in slower sodium release.

In summary, to maximise potential perceived saltiness, salt particles should be designed with small particle size, low density and hydrophobicity and have a particle shape associated with optimal flow properties. Also, the *in vitro* sodium dissolution method used in this study was able to predict key parameters associated with *in vivo* saltiness time-intensity. Future studies should investigate these design rules within a commercial product context and seek to validate the potential for sodium reduction whilst retaining consumer acceptability.

In addition to salt, these physicochemical design rules may apply to new product development and ingredient design of sugar, seasonings and other aligned pharmaceutical and oral care industries, where crystalline structures with controlled dissolution rates are essential for product efficacy.

## Declaration of Competing Interest

The authors declare that they have no known competing financial interests or personal relationships that could have appeared to influence the work reported in this paper.
