# Supplementary material for: Physicochemical design rules for the formulation of novel salt particles with optimised saltiness
Source: Food Chem. 2021 Oct 30;360:129990. doi: 10.1016/j.foodchem.2021.129990 (PMC8223491; doi:10.1016/j.foodchem.2021.129990)
Supplement: Supplementary Data 1 [file mmc1.docx]

**Supplementary materials**

| Supplementary Table 1. Dissolution curve parameters (mean ± SD). T25%, T50%, T75% and T90% are all expressed in seconds (s). Values in the same column with different letters are significantly different (p<0.05). | | | | | | | | |
| --- | --- | --- | --- | --- | --- | --- | --- | --- |
| Dissolution curve parameters | Salt crystals | | | | Modified salt | | | |
|  | RS 106µm | RS 106-425µm | RS 425-600µm | Dendritic | SODA-LO® | FMS <106µm | FMS 106-425µm | FMS 425-600µm |
| Initial Slope (% per second) | 4.0 ± 0.0 BC | 4.5 ± 0.2 AB | 3.3 ± 0.3 DE | 4.2 ± 0.2 B | 4.8 ± 0.1 A | 3.5 ± 0.2 CD | 3.4 ± 0.3 DE | 2.9 ± 0.1 E |
| AUCdiss | 7750 ± 78 CD | 8480 ± 100 A | 7980 ± 170 BC | 8340 ± 120 AB | 8620 ± 30 A | 7650 ± 130 CD | 7630 ± 230 CD | 7500 ± 170 D |
| T25% (s) | 7.3 ± 0.4 CD | 8.0 ± 0.5 CD | 10.7 ± 1.2 AB | 8.0 ± 0.5 CD | 6.7 ± 0.3 D | 8.8 ± 0.3 BC | 10.3 ± 0.4 AB | 11.00 ± 1.00 A |
| T50% (s) | 11.3 ± 1.1 D | 11.6 ± 0.8 D | 16.3 ± 1.4 AB | 12.2 ± 1.4 CD | 9.7 ± 0.3 D | 14.7 ± 1.0 BC | 16.0 ± 0.0 AB | 18.67 ± 0.58 A |
| T75% (s) | 20.5 ± 0.7 CD | 17.7 ± 1.0 AB | 24.8 ± 1.8 BC | 19.3 ± 1.2 D | 14.0 ± 0.0 E | 27.0 ± 2.0 AB | 27.0 ± 1.4 AB | 30.83 ± 2.02 A |
| T90% (s) | 68.5 ± 2.8 A | 26.5 ± 2.5 CD | 37.3 ± 2.8 BC | 31.0 ± 3.0 CD | 21.7 ± 1.2 D | 55.0 ± 5.0 A | 53.5 ± 16.3 AB | 51.67 ± 2.89 AB |

| Supplementary table 2. Pearson correlations between sensory, dissolution measures and particle diameter. Values in bold are different from 0 with a significance level of p=0.05. | | | | | | | |
| --- | --- | --- | --- | --- | --- | --- | --- |
| Variables | Mean particle diameter | T25% | T75% | T50% | T90% | AUC dissolution | Slope dissolution |
| Imax | **-0.83** | **-0.94** | -0.70 | **-0.88** | 0.05 | 0.39 | **0.77** |
| T(First Max) | **0.76** | **0.91** | **0.77** | **0.87** | 0.18 | -0.53 | **-0.79** |
| Rate to Imax | -0.64 | **-0.94** | **-0.83** | **-0.92** | -0.25 | 0.62 | **0.86** |
| T(Last Max) | **0.82** | **0.86** | 0.74 | **0.83** | 0.23 | -0.53 | **-0.77** |
| Max. Duration | 0.75 | 0.62 | 0.55 | 0.62 | 0.25 | -0.43 | -0.60 |
| AUCsensory | **-0.87** | **-0.89** | -0.58 | **-0.79** | 0.21 | 0.23 | 0.69 |
| Area(Max) | -0.07 | -0.39 | -0.19 | -0.33 | 0.37 | -0.04 | 0.22 |
| Area(90% Max) | -0.70 | **-0.87** | -0.66 | **-0.81** | 0.04 | 0.36 | 0.72 |
| T(First 50% Max) | 0.38 | 0.55 | 0.51 | 0.47 | 0.31 | -0.45 | -0.37 |
| T(Last 50% Max) | -0.01 | 0.57 | 0.58 | 0.55 | 0.39 | -0.57 | -0.55 |
| T Start(90% Max) | **0.83** | **0.93** | 0.72 | **0.86** | 0.08 | -0.46 | **-0.81** |
| T Stop(90% Max) | **0.82** | **0.94** | 0.75 | **0.87** | 0.18 | -0.53 | **-0.83** |
| Duration(90% Max) | -0.34 | -0.32 | -0.12 | -0.24 | 0.30 | -0.10 | 0.21 |
| Asc. Start | -0.27 | -0.29 | -0.15 | -0.30 | 0.11 | 0.06 | 0.44 |
| Asc. Stop | **0.76** | **0.91** | **0.77** | **0.87** | 0.18 | -0.53 | **-0.79** |
| Asc. Duration | **0.81** | **0.95** | **0.78** | **0.92** | 0.14 | -0.53 | **-0.89** |
| Desc. Start | **0.82** | **0.86** | 0.74 | **0.83** | 0.23 | -0.53 | **-0.77** |
| Desc. Stop | 0.21 | -0.10 | -0.19 | -0.18 | -0.22 | 0.27 | 0.27 |
| Desc. Duration | -0.26 | -0.57 | -0.59 | -0.64 | -0.33 | 0.55 | 0.69 |
| Desc. Area | **-0.91** | **-0.88** | -0.61 | **-0.81** | 0.13 | 0.28 | 0.71 |
| Desc. Slope | 0.55 | 0.40 | 0.15 | 0.27 | -0.33 | 0.11 | -0.12 |
| Saltiness rating | **-0.83** | **-0.88** | -0.70 | **-0.84** | 0.02 | 0.38 | 0.70 |

| Supplementary table 3. Pearson correlations between physicochemical properties and dissolution parameters. None of the values in are different from 0 with a significance level of p=0.05. | | | | | | | |
| --- | --- | --- | --- | --- | --- | --- | --- |
| Variables | Mean particle diameter | T25% | T75% | T50% | T90% | AUC dissolution | Slope dissolution |
| Mean particle diameter | **1.00** | 0.63 | 0.27 | 0.51 | -0.30 | 0.02 | -0.54 |
| Moisture content (%) | -0.45 | 0.01 | 0.06 | 0.07 | -0.14 | -0.01 | 0.08 |
| Water activity | -0.58 | -0.02 | 0.23 | 0.12 | 0.12 | -0.24 | 0.18 |
| NaCl (%) | 0.19 | -0.46 | -0.68 | -0.55 | -0.40 | 0.62 | 0.22 |
| Bulk Density (g/ml) | 0.70 | -0.07 | -0.39 | -0.21 | -0.47 | 0.51 | 0.05 |
| Tapped density (g/ml) | 0.62 | -0.14 | -0.43 | -0.27 | -0.39 | 0.49 | 0.07 |
| Porosity (%) | **-0.75** | 0.00 | 0.37 | 0.12 | 0.65 | -0.58 | 0.00 |
| Transfer efficiency (%) | **-0.85** | -0.31 | 0.04 | -0.17 | 0.33 | -0.21 | 0.21 |
| Adhesion after packaging test (%) | -0.60 | -0.67 | -0.52 | -0.67 | -0.03 | 0.31 | 0.65 |
| T25% | 0.63 | **1.00** | **0.88** | **0.98** | 0.28 | -0.66 | **-0.91** |
| T75% | 0.27 | **0.88** | **1.00** | **0.95** | 0.63 | **-0.91** | **-0.83** |
| T50% | 0.51 | **0.98** | **0.95** | **1.00** | 0.39 | **-0.76** | **-0.91** |
| T90% | -0.30 | 0.28 | 0.63 | 0.39 | **1.00** | **-0.89** | -0.46 |
| AUC dissolution | 0.02 | -0.66 | **-0.91** | **-0.76** | **-0.89** | **1.00** | **0.73** |
| Slope dissolution | -0.54 | **-0.91** | **-0.83** | **-0.91** | -0.46 | **0.73** | **1.00** |
| Lightness | **-0.90** | -0.33 | 0.09 | -0.17 | 0.52 | -0.35 | 0.29 |
| a* | -0.28 | -0.33 | -0.24 | -0.34 | 0.12 | 0.06 | 0.44 |
| b* | 0.06 | 0.45 | 0.49 | 0.50 | 0.15 | -0.36 | -0.53 |
| Whiteness index | **-0.84** | -0.49 | -0.13 | -0.37 | 0.40 | -0.15 | 0.49 |
